# Toll-Like Receptor Induced Pro-Interleukin-1β and Interleukin-6 in Monocytes Are Lower in Healthy Infants Compared to Adults

**DOI:** 10.1371/journal.pone.0078018

**Published:** 2013-10-25

**Authors:** Daniel H. Libraty, Lei Zhang, Marcia Woda, Luz P. Acosta, AnaMae Obcena, Job D. Brion, Rosario Z. Capeding

**Affiliations:** 1 Division of Infectious Diseases and Immunology, University of Massachusetts Medical School, Worcester, Massachusetts, United States of America; 2 Department of Immunology, Research Institute for Tropical Medicine, Manila, Philippines; 3 Department of Medicine, Research Institute for Tropical Medicine, Manila, Philippines; 4 San Pablo City Health Office, San Pablo, Philippines; 5 Department of Microbiology, Research Institute for Tropical Medicine, Manila, Philippines; Agency for Science, Technology and Research - Singapore Immunology Network, Singapore

## Abstract

Infants have long been known to have higher infectious diseases morbidity and mortality and suboptimal vaccination responses compared to older children and adults. A variety of differences in innate and adaptive immune responses have been described between these two groups. We compared Toll-like receptor (TLR)-induced production of pro-interleukin (IL)-1β, IL-6, and tumor necrosis factor (TNF)-α between 2-month-old infants and adults. TLR 7/8-induced production of pro-IL-1β and IL-6 in monocytes was lower in 2-month-old infants compared to adults. There was no difference in TLR 7/8-induced production of TNF-α. Lower TLR-induced production of pro-IL-1β and IL-6 in innate immune cells during early infancy likely contributes to suboptimal vaccine responses and infectious diseases susceptibility.

## Introduction

Infants have long been known to have higher infectious diseases morbidity and mortality compared to older children and adults. They also have suboptimal vaccination responses to many antigens. One contributing factor has been felt to be differences in the immune responses between infants and older children/adults. A variety of differences in innate and adaptive immune responses have been described between these two groups. The fetal and early neonatal immune system is heavily T-helper 2 (Th2) biased [1], [2]. Toll-like receptor (TLR) responses known to induce Th17 adaptive immune responses peaked at birth and subsequently declined over the next 2 years. TLR responses known to induce anti-viral and Th1 adaptive immune responses were low at birth and slowly increased over the next 2 years [3]. We compared TLR-induced production of pro-interleukin (IL)-1β, IL-6, and tumor necrosis factor (TNF)-α between 2 month old infants and adults. TLR 7/8-induced production of pro-IL-1β and IL-6 in monocytes was lower in 2 month old infants compared to adults.

## Materials and Methods

### Ethics statement

The infant clinical study was approved by the institutional review boards of the Research Institute for Tropical Medicine, Philippines, and the University of Massachusetts Medical School. Mothers and their healthy infants were recruited and enrolled after providing written informed consent.

Peripheral blood mononuclear cells (PBMC) from healthy adult volunteers were collected and isolated using Histopaque® density centrifugation, and cryopreserved. The protocol was approved by the institutional review board of the University of Massachusetts Medical School. Adult volunteers were recruited and enrolled after providing written informed consent.

### Infant clinical study

Details about the infant clinical study protocol have been previously described [4]. Study enrollment began in October 2006 in San Pablo, Philippines. The clinical study is registered at www.clinicaltrials.gov (identifier NCT00377754). Healthy infants and their mothers were enrolled when the infant was between 6–18 weeks old. Peripheral blood mononuclear cells (PBMC) were collected from infants at the first study visit, isolated using Histopaque® density centrifugation, and cryopreserved. Clinical and epidemiological information were also collected at the study visits. Infant weight was measured to the nearest tenth of a kilogram. Infant length was measured to the nearest centimeter. World Health Organization (WHO) body mass index (BMI)-for-age z scores for study infants were determined using the SPSS macro provided by WHO [5]. Infants with missing values or biologically implausible anthropometric z scores were excluded from analyses. Biologically implausible z scores were BMI-for-age z score <−6 or >6.

### Flow cytometry

PBMC were washed with media, and then left unstimulated or stimulated with 1 µM R-848 (Invivogen) ×16 h. The stimulations were done in the presence of 1 µl Brefeldin A (BD Biosciences) ×16 h. Cells were stained with LIVE/DEAD® Fixable Dead Cell Stain Kit (LDA) (Invitrogen), fixed and permeabilized with Cytofix/Cytoperm™ (BD Biosciences), and stained with Abs. Monocytes were identified as LDA-/CD1c-Phycoerythrin (PE)-/CD19-PacOrange-/CD36-Allophycocyanin.Cy7 (APC.Cy7)+/CD123-650 NC-/CD303-APC- and myeloid DCs were identified as LDA-/CD1c^hi^/CD19-/CD36-/CD123-/CD303- (all Abs from eBiosciences). TNF-α, IL-6, and pro-IL-1β production was measured by staining with the respective mAbs (anti-TNF-α-PerCP.Cy5.5, anti-IL-6-Alexa700, anti-IL-1β-PacBlue, BD Biosciences). Cells were analyzed using a FACSAria™ flow cytometer (BD Biosciences). Data was analyzed using FlowJo® software (Treestar).

### Statistical analysis

The SPSS software package (version 20.0) was used for statistical analyses. Comparisons between continuous variables were performed using the non-parametric Mann-Whitney U test. P-values <0.05 were considered significant.

## Results and Discussion

**Figure 1:**
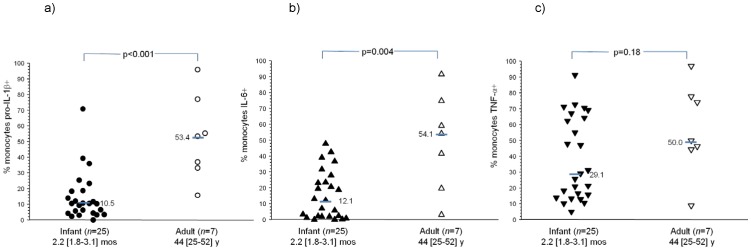
Intracellular cytokine staining in R-848 (1 µM) stimulated monocytes for (a) pro-interleukin (IL)-1β, (b) IL-6, and (c) tumor necrosis factor (TNF)-α. Unstimulated condition is subtracted from each value. Bars are median values. Ages are shown as median [95% confidence interval].

**Figure 2:**
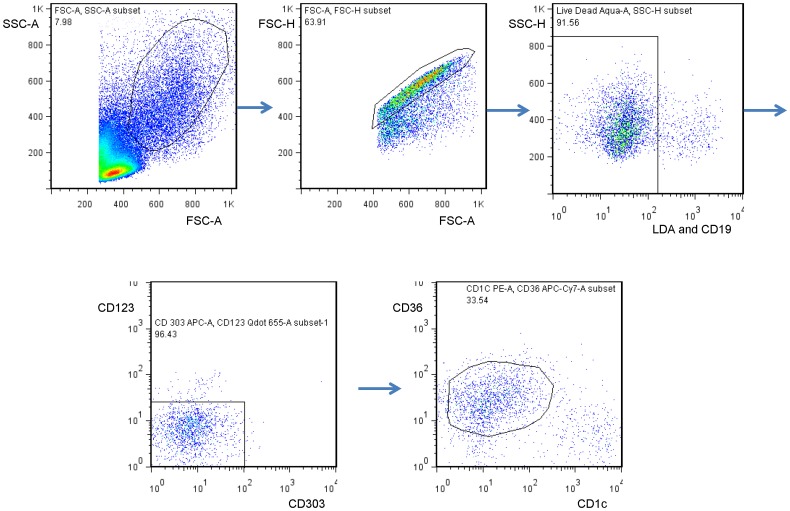
Example of the gating strategy for monocytes- LiveDead Aqua (LDA)-/CD1c-/CD19-/CD36+/CD123-/CD303-.

As part of a prospective study of dengue virus infections during infancy [4], we stimulated PBMC from healthy infants and adults with R-848 and measured intracellular pro-IL-1β, IL-6, and TNF-α by FACS. R-848 is an imidazoquinolone and stimulates human TLR 7/8 [6]. We found that TLR 7/8-induced pro-IL-1β and IL-6 production in monocytes was lower in 2 month old healthy infants (*n* = 25) compared to healthy adults (*n* = 7) (Figure 1). TLR 7/8-induced TNF-α production in monocytes was not different between the two groups. The monocyte gating strategy is shown in Figure 2. Natural killer (NK) cell markers were not utilized and could potentially contaminate the monocyte gate. The vast majority of the infants were well nourished and had a WHO BMI-for-age z score ≥−2 (21/25 (84%)). R-848-induced pro-IL-1β production also trended lower in the circulating myeloid dendritic cells (mDCs) from 2 month old infants compared to adults (% pro-IL-1β+ mDCs upon R-848 (1 µM) stimulation (unstimulated background subtracted): infants (*n* = 33)−10.9 [4.9–14.8], adults (*n* = 5)−19.6 [11.0–27.9], median [95% CI], p = 0.07). R-848-induced IL-6 and TNF-α production in mDCs was not different between infants and adults (data not shown).

In a previous report, pro-IL-1β and IL-6 production in cord blood mononuclear cells upon stimulation with 3M-003, another imidazoquinolone, was similar to adult mononuclear cells and then decreased at 1 and 2 years of age [3]. Our data demonstrates that as early as 2 months of age, TLR 7/8-induced pro-IL-1β and IL-6 production in monocytes is lower than adult responses. The lower TLR-induced production of pro-IL-1β and IL-6 in innate immune cells during early infancy likely contributes to their suboptimal vaccine responses and infectious diseases susceptibility.
